# Autoadjusting-CPAP effect on serum Leptin concentrations in Obstructive Sleep Apnoea patients

**DOI:** 10.1186/1471-2466-8-21

**Published:** 2008-10-01

**Authors:** Marta Drummond, João C Winck, João T Guimarães, Ana C Santos, João Almeida, José A Marques

**Affiliations:** 1 Pulmonology Department, Hospital de São João, Alameda Hernâni Monteiro, 4200-319 Porto, Portugal; 2 Clinical Pathology Department and Biochemistry Department Hospital de São João, Alameda Hernâni Monteiro 4200-319 Porto, Portugal; 3 Epidemiology Department, Hospital de São João, Alameda Hernâni Monteiro, 4200-319 Porto, Portugal

## Abstract

**Background:**

Leptin is an hormone that regulates body weight. Studies have shown increasing leptin concentrations according to body mass index (BMI) and intermittent hypoxia.

Our aim is to evaluate the basal leptin levels in OSA patients and its possible relation to OSA severity, independently of confounders and investigate the Autoadjusting-CPAP effect on leptin values.

**Methods:**

In ninety eight male patients with moderate to severe OSA leptin serum levels were evaluated before therapy, 9 days and 6 months after therapy.

**Results:**

In this group mean age was 55.3 years, mean BMI was 33.2 Kg/m2 and mean Apnoea- Hypopnea Index (AHI) was 51.7/h. Mean basal serum leptin value was 12.1 ug/L. Univariate analysis showed a significant correlation between serum leptin values and BMI (R = 0.68; p < 0.001), waist-hip ratio (R = 0.283; p = 0.004) and AHI (R = 0.198; p = 0.048); in stepwise multiple regression analysis only BMI (p < 0.001) was a predictor of serum leptin values.

One week after therapy, mean leptin serum level decreased to 11.0 ug/L and 6 months after it was 11.4 ug/L. (p = 0.56 and p = 0.387, respectively)

**Conclusion:**

Baseline leptin serum levels positively correlate with BMI, fat distributioand OSA severity.

BMI is the only predictor of basal leptin levels.

Treatment with Autoadjusting-CPAP has a small effect on leptin levels.

## Background

Obstructive Sleep Apnoea (OSA) is a common disorder with a prevalence of 2% to 4% in middle-aged adults [1].

The association between obesity and OSA is strong and well described. Obesity is a major risk factor for OSA, which occurs in up to 50% of obese men [1-6]. In addition, approximately 70% patients with OSA are obese [7]. It is known that every 10-Kg increment in body weight increases OSA risk twofold [8].

Leptin is a hormone with well Known functions concerning body composition, energy homeostasis and feeding behavior in humans [9-11]. It is an 167-amino acids peptide hormone produced predominantly in white adipose tissue [9-11]. Leptin circulates in the plasma in a free-form state or bound to leptin-binding proteins [11]. It acts by binding to specific receptors in the hypothalamus to decrease appetite and increase energy expenditure [9-14]. Leptin inhibits the synthesis of hypotalamic neuropeptide y (NPY), a potent stimulator of food intake, furthermore, downregulation of NPY increases sympathetic nervous system outflow enhancing energy expenditure [9-18].

In fact, leptin informs the brain about the size of adipose stores and has been thought to be the obesity hormone regulator [10].

The dramatic weight reduction observed in *ob/ob *mice in result to leptin administration, raised expectations that human obesity might also be a leptin deficient state treatable with exogenous leptin administration [19,20]. However, unlike the tight relationship present between obesity and serum leptin levels in mice, human obesity seems to be associated with more possible derrangements from leptin deficiency to leptin resistance [21,22].

Leptin levels increase exponentially with increasing body weight [11,22,23] and previous studies [22,24,25] have reported a significant correlation between measures of body fat and circulating concentrations of leptin in adults.

Under experimental conditions, intermittent hypoxia stimulates leptin production and a number of studies have demonstrated that serum leptin levels are elevated in OSA patients, independently of obesity [18,26-32] despite some couldn't find that relationship when controlled for body fat [12,33,34]. If OSA is truly associated with increased leptin serum levels independent of obesity, the causal link remains unclear. Rodent studies [35,36] provided evidence for a leptin influence in breathing stability, and thus predisposing individuals to develop OSA. In alternative, OSA may be a cause of elevated leptin serum levels through the effects of hypoxemia, sleep fragmentation or heightened sympathetic activity. Data showing leptin serum levels reduction after CPAP nocturnal usage in OSA patients [32,34,37-41] reinforces this idea.

The present study was conducted to evaluate the basal serum leptin levels in OSA patients and its possible relation to OSA severity. This study has also the purpose of understanding the Autoadjusting-CPAP effect on leptin circulating levels after short and long term treatment, as this effect has not been outlined yet, and the automated pressure can play a different role in leptin levels variation than that of fixed CPAP. Thus we try to clarify the relationship between leptin and sleep apneic activity, independently of confounders.

## Methods

### Study design

This trial was designed as a prospective study. All patients gave written informed consent to participate in the trial. The study protocol was approved by the Hospital Ethics Committe and the study was performed in accordance with the guidelines of the Declaration of Helsinki and its current revision.

### Subjects

One hundred and two male consecutive patients referred for suspected sleep disordered breathing to our Sleep Disordered Breathing Clinic were included in the study, between February 2005 and March 2006.

All patients presented moderate/severe OSA (AHI > 20/h) confirmed by domiciliary sleep study.

Exclusion criteria were stablished previously: neoplastic diseases, systemic inflammatory chronic diseases, active infectious diseases, systemic long term corticotherapy and female gender.

All but four patients concluded the study (n = 98). Those who failed to conclude the protocol were due to Autoadjusting-CPAP intolerance.

### Study procedures

An overnight sleep study was performed using a five-channel recording device (*Alphascreen; Vyasis*). This device produces a computorized recording of variations in oronasal airflow (measured by nasal cannula), body position, wrist actimetry, pulse rate and arterial oxygen saturation (measured by finger pulse oximetry). The device estimates the total sleep time from the wrist actimetry registry, eliminating those periods with high activity. It automatically calculates the number of apnoeas plus hypopnoeas per hour of estimated sleep time (automatic respiratory disturbance index) and it also provides information of desaturations > 4% per hour of estimated sleep time and the cumulative percentages of sleep time under 90% oxygen saturation. In all cases, sleep technicians carried out a manual analysis of the recordings, by counting apnoea (events of airflow cessation lasting for at least 10 seconds) and hypopnoea episodes (events of airflow reduction to 20 to 50% of the previously observed lasting for at least 10 seconds, joined with a 4% dip in oxygen saturation and/or an arousal), dividing the total number of these episodes by the sleep time in hours, thus obtaining the manual apnoea/hypopnoea index (AHI) according to established criteria [42].

Auto-CPAP therapy was prescribed to all patients with a minimum pressure of 4 and a maximum pressure of 15 cmH20.

24-hour Ambulatory Blood Pressure (*Spacelab, Inc 90207 Neural*) was performed in all but 3 patients who refused the examination as they considered the arm disconfort intolerable.

Fasting morning venous blood samples were collected between 8–10 a.m. before treatment, one week after and finally 6 months after the treatment initiation.

Blood samples were immediately sent to the laboratory for estimation of glucose and lipids, while a specimen of clotted blood was centrifuged at 4000 g for 20 min for serum, which was stored at -26°C in eppendorfs^® ^until leptin analysis was performed.

Serum leptin concentrations were measured in duplicates with a highly sensitive radioimmunoassay [43] (Linco Res; St. Louis MO). The sensitivity of this assay was 0,5 ng/mL, the specificity was 100% and the interassay coefficient of variation was 4,6%.

### Statistical Analysis

Data were analyzed using SPSS, release 14.0, and described as mean values and their respective standard deviation for normally, or as median values and corresponding 25^th ^and 75^th ^centiles for clearly non-normally distributed variables. Counts and proportions are reported for categorical variables. Proportions were compared using Chi-square test or *Fisher's *exact test whenever appropriate. Means and mean differences of leptin levels between the second and the first period of observations as well as between the third and the first periods of observation were compared by ANOVA or by non-parametric equivalent.

Linear multiple regression models were used to estimate the magnitude of the association between leptin levels and the studied determinants.

## Results

### Sample Characteristics

Table 1 summarizes the sample characteristics.

**Table 1:** Characteristics of the study group

	**Study group**
**Age (years)**	55.3 ± 10.7
**BMI (Kg/m2)**	33.2 ± 5.0
**Waist-to-hip ratio**	1 ± 0.1

Table 2 shows the patients distribution according to Bray's obesity categories [44].

**Table 2:** Patients distribution according to Bray's obesity categories

	***Class 0*** (*BMI: 20–25*)	***Class I*** (*BMI: 25–30*)	***Class II*** (*BMI: 30–35*)	***Class III*** (*BMI: 35–40*)	***Class IV*** (*BMI > 40*)
Patients	3.8%	23.6%	36.8%	30.2%	6.7%

During the 6 months of the study, patients couldn't loose significant weight (mean baseline weight = 94.4 Kg; mean final weight = 94.1 Kg; p = 0.545).

Table 3 summarizes sleep study information.

**Table 3:** Sleep studies information

	***Study group***
**AHI**	51.7 ± 21.3
**Desaturation index**	86.3 ± 5.3
**Lowest 02 (%)**	70.8 ± 9.7

Mean 02 (%)	86.3 ± 5.3

### Habits and Comorbidities

In this population 42.5% patients were non-smokers; 39.6% former- smokers (> 1 year without smoking habits) and 17.9% had active smoking habits.

Arterial Hypertension (AH) was found, according to 24-hour ambulatory blood pressure results, in 47.2% cases, being 2.8% nocturnal AH.

Congestive heart failure was present in 9.4% patients, according to clinical symptoms and used medication. History of stroke, acute myocardial infarction (AMI) and angina was present in 15.1%, 7.5% and 1.9% patients, respectively.

Blood analysis showed that 75.5% patients had high serum lipid values (total cholesterol > 2.00 g/L; LDL > 1.30 g/L; triglicerides > 1.50 g/L) and 34.9% showed glucose intolerance (fasting glucose > 1.15 g/L; HgA1c > 6%).

### Leptin

Leptin serum levels are showed in table 4.

**Table 4:** Leptin serum levels

	***Basal***	***One week after therapy***	***6 months after therapy***
**Leptin Serum Levels (ug/L)**	12.1 ± 12.2	11 ± 10.7	11.4 ± 10.6

Leptin variation significance (p) according to baseline		0.56	0.387

Sixty percent of patients presented elevated basal leptin levels. The maximum value was 56.0 ug/L. The patients with elevated basal serum leptin levels had a higher BMI than the others (p < 0,001) but did not present a central fat distribution (p = 0.052).

Leptin levels decrease after short and long-term Auto-CPAP therapy were not statistically significant.

### Confounders analysis

We analyzed whether age, BMI, fat distribution (waist-hip ratio), OSA severity (AHI, dessaturation index, minimum 02 saturation), serum lipids and glucose intolerance could be correlated with basal serum leptin levels or not.

An univariate analysis showed a significant correlation between serum leptin values and BMI (R = 0.68; p < 0.001), waist-hip ratio (R = 0.283; p = 0.004) and AHI (R = 0.198; p = 0.048).

In stepwise multiple regression analysis only BMI (p < 0.001) was a predictor of basal serum leptin values and AHI was not found to be significant.

### Auto-CPAP compliance

Compliance with Auto-CPAP was good but it was not related to leptin decrease (table 5). Pressure on 90% nighttime decreased significantly during the study (mean baseline p90 = 10.8 cmH20; mean final p90 = 10.1; p < 0,001) and the mean residual AHI was 2.7/h ± 1.7.

**Table 5:** Serum Leptin variation and Auto-CPAP usage

***Spearman's test***	***% Auto-CPAP days of usage***	***Total Auto-CPAP days of usage***	***Hours per night of Auto-CPAP usage***
**Mean**	91.27 ± 20.45	171.2 ± 44.9	5.76 ± 1.59

Serum Leptin variation significance (p)	0.184	0.247	0.795

## Discussion

Obesity is the major factor regulating circulating leptin [24,25,45] which is also influenced by gender and age [23,46].

In this study, the authors found a strong and highly significant positive correlation between obesity (BMI) and serum leptin levels at baseline (p < 0.001) as others have demonstrated [11,22-25,45,47].

Several previous studies have reported increased plasma leptin levels in patients with OSA [29,37,42,48], however the relationship between leptin and OSA is far from being resolved mainly because the potential confounding role of obesity.

We found that leptin levels are clearly elevated in OSA patients (mean basal value = 12.1 ± 12.2 ug/L; men normal range: 3.8 ± 1.8 ug/L) but these levels do not correlate with OSA severity when considering confounders as obesity and fat distribution. In this study, after performing multiple regression analysis, the BMI was the only predictor of baseline leptin serum levels. Also, some authors could not find correlation between the OSA severity and the leptin values when corrected for obesity [12,33] but others did [26-30]. The latter studies included lower number of patients when compared to the former and we could speculate about the importance of the sample magnitude to acquire statistical significance in differences encountered.

We found that Auto-CPAP reduced only marginally leptin plasma levels both in short and long term treatment (6 months). This may seem in contrast with previous studies that have generally shown that leptin levels decrease significantly with CPAP [36-41,48], but these changes were generally small in absolute amount and not very different from those determined here. Furthermore, although clinical and physiological efficacy of Auto-CPAP is equivalent to fixed CPAP [18,32] and despite the conclusions of numerous studies about Auto-CPAP efficacy [49-55] in controlling respiratory events and hypoxemia that is presumably implicated in plasma leptin levels [18,32], we cannot exclude that nighttime pressure variation with Auto-CPAP can affect leptin levels in a different way than fixed CPAP does.

In summary, taking all these observations into account our findings suggest that the increased leptin levels described so far in patients with OSA is mostly associated with obesity and not with the disease itself or its severity; accordingly short and long term treatment with CPAP has a small effect on leptin plasma levels and Auto-CPAP therapy compliance is not related with the leptin values decrease.

As our patients didn't demonstrate a significant weight loss, this variable could not be studied as a potential influencing factor in leptin levels.

Some characteristics of our study deserve comment.

In order to get a more homogenous sample in this study, the authors decided not to include women because of gender effect on serum leptin circulating values. Leptin serum levels are significantly higher in pre- and post-menopausal female when compared to male, even after correction for differences in body composition [23,46]. This sexual dimorphism is apparently due to estrogen and progesterone effects and eventually to an androgen supressive effect on leptin [44].

The authors do not consider the OSA diagnosis based on a domiciliary sleep study a limitation of the present study as this diagnosis tool has already been compared to polysomnography showing to be a viable, accurate, satisfactory, useful and cost effective way of diagnosing OSA [56,57].

The lack of a control group can be seen as a limitation of this study. Nevertheless we could demonstrate that OSA patients have elevated serum leptin levels and we could establish a significant positive correlation between BMI and leptin levels.

## Conclusion

OSA patients show elevated serum plasma levels.

BMI constituted itself as the only predictor of baseline leptin levels.

Short- and long-term Auto-CPAP therapy produces a small and not significant reduction in plasma leptin levels.

## Competing interests

The authors declare that they have no competing interests.

## Authors' contributions

MD conceived the study, participated in its design, data acquisition, coordination and drafted the manuscript. JCW conceived the study, participated in its design and data acquisition. JTG coordinated the chemical analysis. ACS performed the statistical analysis. JA participated in data acquisition. JAM conceived the study and participated in its design. All the authors read and approved the final manuscript.

## Pre-publication history

The pre-publication history for this paper can be accessed here:


